# Real-world evaluation of the FluoroType MTBDR 2.0 assay for detection of rifampicin and isoniazid resistance-conferring mutations in *Mycobacterium tuberculosis* complex clinical isolates

**DOI:** 10.1128/spectrum.01880-26

**Published:** 2026-09-18

**Authors:** Fabiane Maria de Almeida Ferreira, Angela Pires Brandão, Thiago Brobio Massanti, Antonio Carlos da Silva Rego, Lucilaine Ferrazoli, Erica Chimara, Rosângela Siqueira de Oliveira, Juliana Maira Watanabe Pinhata

**Affiliations:** 1 Núcleo de Tuberculose e Micobacterioses, Centro de Bacteriologia, Instituto Adolfo Lutz (IAL) 89119, São Paulo, São Paulo, Brazil; 2 Instituto Oswaldo Cruz, Fundação Oswaldo Cruz (FIOCRUZ) 37903, Rio de Janeiro, Rio de Janeiro, Brazil; 3 Mobius Lifescience, Pinhais, Paraná, Brazil; Indiana University School of Medicine, Indianapolis, Indiana, USA; University of Pennsylvania, Philadelphia, Pennsylvania

**Keywords:** drug resistance, FluoroType MTBDR, line probe assay, multidrug-resistant tuberculosis, *Mycobacterium tuberculosis*

## Abstract

Drug-resistant tuberculosis (TB) remains a major global health challenge, particularly in high-burden settings, where rapid detection of resistance to rifampicin (RIF) and isoniazid (INH) is essential for timely treatment. The FluoroType MTBDR assay is an automated real-time PCR platform for simultaneous detection of *Mycobacterium tuberculosis* complex (MTBC) and resistance to RIF and INH. This study aimed to evaluate the performance of FluoroType using clinical MTBC isolates under routine laboratory conditions in a reference setting. The assay was prospectively assessed in comparison with the GenoType MTBDR*plus* line probe assay (LPA) using 1,841 clinical isolates, with discordant results resolved by Sanger sequencing. For MTBC detection, FluoroType achieved 100% sensitivity (1,807/1,807), 88.2% specificity (30/34), and 99.8% overall accuracy. FluoroType demonstrated 99.9% (1,683/1,685) sensitivity in detecting wild-type isolates. For resistance detection, FluoroType showed 95.5% sensitivity for RIF (42/44) and 94.4% for INH (34/36). Among multidrug-resistant isolates, sensitivity was 85.4% (35/41). Discordances were mainly associated with heteroresistance, and uncommon or synonymous mutations. Importantly, limitations in identifying specific mutations highlight the need for cautious interpretation and, when necessary, confirmatory sequencing. Overall, these findings support FluoroType as a rapid, high-throughput, and reliable tool for MTBC detection and drug resistance profiling in reference laboratories.

**IMPORTANCE:**

This study is the first to prospectively evaluate the FluoroType MTBDR assay performance using a large collection of clinical isolates under routine conditions in a high-burden tuberculosis (TB) reference laboratory. It assesses detection of *Mycobacterium tuberculosis* complex and resistance to rifampicin and isoniazid. Drug-resistant TB remains a major global health challenge, requiring rapid and reliable diagnostic tools. FluoroType demonstrated high accuracy and operational advantages over the line probe assay (LPA), including a streamlined workflow and reduced turnaround time for high-throughput settings. Importantly, this study highlights limitations shared by molecular assays, particularly in detecting and interpreting heteroresistance, uncommon mutations, and synonymous rpoB variants, which may lead to discrepant resistance classification across platforms. These findings underscore the need for cautious interpretation of molecular results and sequencing to resolve clinically relevant discrepancies. Overall, FluoroType is a valuable addition to TB diagnostic algorithms, but results should be interpreted in the context of assay-specific limitations.

## INTRODUCTION

Brazil is among the 30 high-burden countries for both tuberculosis (TB) and TB-HIV coinfection, and is therefore considered a priority setting for global TB control (1). In 2025, the country reported 84,368 new TB cases, corresponding to an incidence rate of 39.5 cases per 100,000 population. In the same year, 6,376 TB-related deaths were recorded, resulting in a mortality rate of 3.0 per 100,000 population (2).

Approximately 24% of all TB cases reported annually in Brazil occur in the state of São Paulo (SP). In 2025, SP accounted for 19,891 notified cases, with an incidence rate of 43.2 per 100,000 population (2). Regarding drug resistance, Brazil reported 1,145 new cases of drug-resistant TB (DR-TB) in 2025, of which 747 (65.2%) were classified as rifampicin-resistant (RR) or multidrug-resistant TB (MDR-TB) (2), defined as resistance of *Mycobacterium tuberculosis* complex (MTBC) to at least rifampicin (RIF) and isoniazid (INH).

Phenotypic drug susceptibility testing (pDST) based on culture remains the reference standard for detecting MTBC resistance to the majority of anti-TB drugs. However, this approach is time-consuming and labor-intensive due to the slow growth rate of the bacillus, requiring up to 8 weeks for colony formation on solid media (3). Automated liquid culture systems, such as the BD BACTEC MGIT 960 (Becton, Dickinson and Company, USA), have reduced turnaround times to approximately 2 weeks (4). Nevertheless, these methods still depend on bacterial growth, which delays clinical decision-making.

To overcome these limitations, molecular diagnostic tests have been developed to enable rapid detection of drug resistance by identifying mutations associated with resistance in MTBC (5). In recent years, automated real-time PCR (qPCR)-based assays using centralized platforms have been increasingly adopted in reference laboratories, allowing simultaneous diagnosis of TB and detection of resistance to key drugs. Several of these technologies are currently recommended by the World Health Organization (WHO) for the initial diagnosis of TB and rapid detection of resistance to RIF and INH directly from pulmonary specimens (6).

The FluoroType MTBDR assay (FluoroType) (Bruker-Hain Lifescience, Germany) is one such WHO-endorsed technology. Unlike conventional line probe assays (LPAs), FluoroType combines real-time PCR amplification with automated fluorescence detection and result interpretation, eliminating the post-PCR hybridization step. The assay can be applied directly to NALC-NaOH-decontaminated sputum samples or to MTBC isolates obtained from solid or liquid (MGIT) culture media (7). Its high-throughput 96-well format allows the simultaneous analysis of up to 94 clinical samples per run (plus controls), making it well suited for routine testing in high-volume reference laboratories. MTBC detection is based on identification of the *rpoB* gene and/or the *IS6110* insertion sequence. RIF resistance is determined by mutations within the 81 bp rifampicin resistance-determining region (RRDR) of the *rpoB* gene, while INH resistance is assessed through mutations at codon 315 of the *katG* gene and in the promoter region of the *inhA* gene (8, 9). The assay is capable of detecting up to 45 mutation types, including point mutations, insertions, deletions, silent mutations, and combinations thereof, making it the only WHO-endorsed qPCR assay capable of identifying the specific resistance-associated mutation(s) rather than simply reporting resistance, with a reported detection limit of 20 CFU/mL (10).

The evaluation and incorporation of novel diagnostic technologies are key responsibilities of reference laboratories, particularly in high-burden settings. In this context, the FluoroType assay represents a promising tool due to its high-throughput capacity and potential for rapid and accurate detection of drug resistance. Therefore, this study aimed to evaluate the performance of FluoroType for detecting rifampicin and isoniazid resistance in clinical MTBC isolates under routine conditions in a reference laboratory.

## MATERIALS AND METHODS

### Sample size calculation

Sample size was estimated to prospectively evaluate the diagnostic performance of FluoroType in our setting, based on routine laboratory data generated using the GenoType MTBDR*plus* v.2 (Bruker-Hain Lifescience) LPA.

The minimum number of drug-resistant isolates required was calculated using the formula: *N* ≥ *Z*² × *P* × (1 − *P*)/*D*², where *N* is the number of resistant samples, *Z* is the standard normal deviate (1.96 for a 95% confidence interval), *P* is the expected sensitivity (0.89 for INH and 0.93 for RIF), and *D* is the desired precision (0.1) (11). Based on this approach, a minimum of 38 isoniazid-resistant (INH-R) and 25 RR isolates were required.

To estimate the total number of MTBC cultures required, we considered (i) the prevalence of resistance to RIF (3.0%) and INH (2.3%) among MTBC isolates previously tested using the LPAs in our setting; and (ii) an approximate 4% rate of MTBC-negative or invalid results. Based on these parameters, we estimated that 1,701 MTBC cultures would need to be tested to obtain the required number of INH-R and RR isolates.

For MDR-TB, assuming an expected sensitivity of 0.89, the same formula indicated that 38 MDR isolates would be required. However, due to the low prevalence of MDR-TB in our setting (0.6%), a purely prospective design would require 6,519 isolates to reach this target. To address this limitation, we supplemented the prospective sample with all MDR isolates identified by the LPA in our laboratory between January and December 2024, resulting in a total of 41 MDR isolates included in the analysis.

### Clinical isolates

Clinical mycobacterial isolates grown in MGIT or on solid culture media are routinely referred to our laboratory by TB network laboratories performing culture in the state of SP for species identification and DST. Upon receipt, isolates are initially evaluated based on growth and colony characteristics to allow presumptive differentiation between MTBC and non-tuberculous mycobacteria (NTM) (12). To ensure independence of observations, only one isolate per patient was included in the analysis.

Isolates prospectively tested using the LPA between April and July 2024 were included in the study. For the evaluation of MDR, all isolates identified as MDR in 2024 were additionally included. For LPA testing, DNA was extracted shortly after isolate receipt according to routine laboratory procedures. Following LPA testing, isolates grown in MGIT were stored at 35°C ± 2°C, whereas those cultivated on solid media were maintained at 2°C–8°C until DNA extraction for testing with FluoroType.

### GenoType MTBDR*plus* LPA

DNA extraction, PCR amplification, and hybridization of PCR products to nitrocellulose strips were performed according to the manufacturer’s instructions (13). Hybridization was carried out using the GT-Blot 48 (Bruker-Hain Lifescience), and results were interpreted using the GenoScan software. After verification by a trained professional, results were released into the laboratory system according to routine procedures.

An inconclusive result was defined as the presence of a positive *M. tuberculosis* complex (TUB) band—indicating MTBC detection—in combination with faint wild-type (WT) bands for resistance-associated genes and absence of mutation (MUT) bands, suggestive of low bacterial load and impaired resistance detection. All isolates with inconclusive results were retested.

Heteroresistance was defined as the simultaneous hybridization of both a WT probe and its corresponding MUT probe, indicating the presence of mixed susceptible and resistant MTBC populations within the same isolate.

Isolates negative for MTBC by the LPA were further evaluated using the Bioline TB Ag MPT64 test (Abbott, USA) for MTBC confirmation. Samples remaining negative were subsequently analyzed with the GenoType *Mycobacterium* CM v.2 (Bruker-Hain Lifescience) for NTM identification. Isolates not identified to the species level were further characterized using the in-house PRA-*hsp65* method (14).

### FluoroType MTBDR

The assay was performed according to the manufacturer’s instructions (7). DNA was extracted from cultured isolates using the FluoroLyse kit (Bruker-Hain Lifescience).

FluoroType testing was carried out using the FluoroCycler 96 instrument. PCR mixtures were freshly prepared by combining 6 µL of Amplification Mix A and 14 µL of Amplification Mix B, followed by the addition of 20 µL of extracted DNA. Positive and negative controls were included in each run (15). Reaction mixtures were immediately loaded into the instrument.

Following the run, results were automatically analyzed using the FluoroSoftware, which validates internal controls, confirms MTBC identification, and identifies mutations in the *rpoB*, *katG*, and *inhA* genes. The software reports unknown signatures (distinct from wild-type patterns) as MUT (undifferentiated mutations) and classifies results as IND (indeterminate) when the distinction between wild-type and mutant profiles is not possible. In cases of failure in amplification or control validation, results are classified as invalid (15).

Isolates with invalid or indeterminate FluoroType results were retested, and discordant isolates between FluoroType and the LPA were retested prior to Sanger sequencing.

### Gene sequencing

Following the methodology described by Pinhata et al. (16), isolates showing discordant results between FluoroType and the LPA, as well as those with inferred resistance-associated mutations identified by the LPA (i.e., absence of hybridization to one or more wild-type probes without hybridization to a corresponding mutation-specific probe), were subjected to Sanger sequencing.

The *inhA* promoter region (positions −168 to +80 relative to the start codon) was amplified using primers inhA-1 and inhA-2 (17). The *katG* gene was amplified using primer pairs katG-P4 through katG-P8, covering positions −135 to 2,202, including an additional 431 nucleotides downstream (18). A 350 bp fragment of the *rpoB* gene, encompassing the RRDR (positions 1,184 to 1,533), was amplified using primers rpoB-1 and rpoB-2 (19).

PCR reactions were performed using the GoTaq G2 Flexi DNA Polymerase kit (Promega, USA). Each 25 µL reaction contained 1× colorless buffer, 200 µM dNTPs, 1.5 mM MgCl₂ for *inhA* and *katG* (1.75 mM for *rpoB*), 5 pmol of each primer for *inhA* and *katG* (10 pmol for *rpoB*), 0.75 U of Taq DNA polymerase, approximately 50 ng (2.6 µL) of template DNA, and nuclease-free water (Qiagen, USA) (16). Thermal cycling conditions included an initial denaturation at 94°C for 5 min, followed by 40 cycles of denaturation at 94°C for 1 min, annealing at 64°C (*inhA*), 62°C (*katG*), or 60°C (*rpoB*) for 1 min, and extension at 72°C for 1 min, followed by a final extension at 72°C for 10 min (16).

PCR products were purified using ExoSAP-IT (Affymetrix, USA) and sequenced on an ABI 3130xl Genetic Analyzer (Applied Biosystems, USA) using the BigDye Terminator v3.1 Cycle Sequencing Kit. Sequence chromatograms were analyzed using BioEdit v7.2.5 and aligned with reference sequences using the MUBII-TB-DB and BLAST tools (20).

## RESULTS

A total of 1,841 cultures presumptively identified as MTBC were analyzed using both the LPA and FluoroType, and the results were compared (Table 1). For MTBC detection, the LPA identified 1,807/1,841 isolates (98.2%) as MTBC, and FluoroType showed 100% concordance with the LPA for these MTBC-positive isolates. Among the remaining 34 isolates (1.8%) classified as non-MTBC by the LPA, 30 were also classified as non-MTBC by FluoroType, whereas four were identified as MTBC-positive. Overall, when compared with the LPA, FluoroType demonstrated a sensitivity of 100%, specificity of 88.2%, and overall accuracy of 99.8% for MTBC detection (Table 2).

**TABLE 1:** Results of the LPA GenoType MTBDR*plus* and FluoroType MTBDR for mycobacterial isolates tested prospectively^*a*^

LPA GenoType MTBDR*plus*							
FluoroType MTBDR	Wild type (WT)	RIF-R	INH-R	MDR	MTBC positive, INH WT, RIF inconclusive	Non-MTBC	Total
Wild type (WT)	1,683	1^*c*^	1^*e*^	0	1	1^*i*^	1,687
RIF-R	1	42^*b*^	0	2	0	0	45
INH-R	0	0	34^*f*^	4^*h*^	0	0	38
MDR	0	0	0	35	0	0	35
MTBC positive, RIF and INH resistance indeterminate	0	0	0	0	0	3^*j*^	3
MTBC positive, RIF WT, INH indeterminate	1	0	1^*g*^	0	0	0	2
MTBC positive, INH WT, RIF indeterminate	0	1^*d*^	0	0	0	0	1
Non-MTBC	0	0	0	0	0	30	30
Total	1,685	44	36	41	1	34	1,841

^*a*^ RIF-R, rifampicin-resistant; INH-R, isoniazid-resistant; MDR, multidrug-resistant; MTBC, *Mycobacterium tuberculosis *complex; WT, wild type; non-MTBC, negative for MTBC by the assays.

^*b*^ 5 with silent F433= mutation by FluoroType and sequencing.

^*c*^ *rpoB *heteroresistant (S450L+WT8) by LPA and wild type by sequencing.

^*d*^ Isolate with inferred *rpoB *mutation (WT1 negative) by LPA.

^*e*^ *katG *heteroresistant (S315T1+WT positive) by LPA.

^*f*^ 2 isolates *katG *heteroresistant (S315T1+WT positive) by LPA.

^*g*^ *inhA *heteroresistant (C-15T+WT1 positive) by LPA.

^*h*^ 2 with RIF resistance indeterminate.

^*i*^ Patient notified with TB, treated, and cured.

^*j*^ 1 patient not notified with TB.

**TABLE 2:** FluoroType MTBDR assay performance in comparison to the LPA GenoType MTBDR*plus*

FluoroType results	Sensitivity, % (*n*/*N*)	Specificity, % (*n*/*N*)	Accuracy, % (*n*/*N*)
*M. tuberculosis* complex detection	100.0 (1,807/1,807)	88.2 (30/34)	99.8 (1,837/1,841)
Wild type (no mutations detected)	99.9 (1,683/1,685)	98.7 (154/156)	99.8 (1,837/1,841)
Rifampicin resistance (RIF-R)	95.5 (42/44)	99.8 (1,758/1,762)	99.7 (1,800/1,806)
Isoniazid resistance (INH-R)	94.4 (34/36)	100.0 (1,770/1,770)	99.9 (1,804/1,806)
Multidrug-resistant (MDR)	85.4 (35/41)	100.0 (1,765/1,765)	99.7 (1,800/1,806)

The four discordant isolates (non-MTBC by LPA but MTBC-positive by FluoroType) were later identified as NTM (*M. abscessus* subsp. *abscessus*, *M. doricum*, *M. fortuitum*, and *M. gordonae*). Notably, three of the four patients from whom these NTM were isolated were diagnosed with and treated for TB.

For the evaluation of resistance detection, 1,806 MTBC isolates with valid LPA results were included; one isolate was excluded due to an inconclusive RIF result. Among the 1,685 isolates classified as wild type by LPA, FluoroType showed concordant results for 1,683, corresponding to a sensitivity of 99.9%. The two discordant isolates comprised one classified as RIF wild-type and *katG* indeterminate by FluoroType but wild type by sequencing, and another in which FluoroType detected an *rpoB* mutation (R448K), whereas sequencing indicated a wild-type RRDR, although a P454S mutation located outside the RRDR was identified (Tables 1 to 3).

**TABLE 3:** Results of LPA GenoType MTBDR*plus*, FluoroType MTBDR, and gene sequencing for all 1,841 mycobacterial isolates tested^*a*^

LPA MTBDR*plus* result	Mutations detected by LPA MTBDR*plus*	Mutations detected by FluoroType	Gene sequencing result	No. of isolates
No mutations found (*N* =1,685)	*rpoB_*WT; *katG*_WT; *inhA*_WT	*rpoB*_WT; *katG*_WT; *inhA*_WT	ND	1,683
		*rpoB_*WT; ***katG_*****indeterminate**; *inhA*_WT	*katG_*WT	1
		***rpoB*_R448K**; *katG*_WT; *inhA*_WT	*rpoB*_WT RRDR; *rpoB_*P454S, 1360 ccc>tcc (out of RRDR)	1
RIF-R (*N* = 44)	*rpoB_*H445D; *katG*_WT; *inhA*_WT	*rpoB_*H445D/S; *katG*_WT; *inhA*_WT	ND	1
	*rpoB_*S450L; *katG*_WT; *inhA*_WT	*rpoB_*S450L/W; *katG*_WT; *inhA*_WT	ND	5
	*rpoB_*WT2 absent [inferred]; *katG*_WT; *inhA*_WT	*rpoB*_L430P; *katG*_WT; *inhA*_WT	*rpoB_*L430P, 1289 ctg>ccg (*n* = 3); L430R, 1289 ctg>cgg (*n* = 1)	4
	*rpoB_*WT3 absent [inferred]; *katG_*WT; *inhA_*WT	*rpoB*_F433=; *katG*_WT; *inhA*_WT	*rpoB_*F433=, 1299 ttc>ttt	5
	*rpoB_*WT3+4 absent [inferred]; *katG_*WT; *inhA_*WT	*rpoB*_D435G; *katG*_WT; *inhA*_WT	*rpoB_*D435G, 1304 cag>ggc	1
	*rpoB_*WT5 absent [inferred]; *katG_*WT; *inhA_*WT	***rpoB_*H445C**; *katG*_WT; *inhA*_WT	*rpoB*_P439L, 1316 ccg>ctg	1
	*rpoB_*WT7 absent [inferred]; *katG_*WT; *inhA_*WT	*rpoB_*H445N; *katG*_WT; *inhA*_WT	*rpoB*_H445N, 1333 cac>aac	24
		***rpoB*_S441L**; *katG*_WT; *inhA*_WT	*rpoB_*T444=, 1322 acc>act	1
	*rpoB_* S450L+WT8 [HR]; *katG*_WT; *inhA*_WT	***rpoB*_WT**; *katG*_WT; *inhA*_WT	*rpoB*_WT	1
	*rpoB_*WT1 absent [inferred]; *katG*_WT; *inhA*_WT	***rpoB_*indeterminate**; *katG*_WT; *inhA*_WT	*rpoB*_T427=, 1281 acc>act	1
RIF inconclusive, INH susceptible (*N* = 1)	*rpoB_*WT6+7+8 absent [low DNA load]; *katG*_WT; *inhA*_WT^*c*^	*rpoB*_WT	*rpoB*_WT	1
INH-R (*N* = 36)	*rpoB_*WT; *katG*_S315T1; *inhA*_WT	*rpoB_*WT; *katG*_S315T1; *inhA*_WT	ND	8
	*rpoB_*WT; *katG*_WT; *inhA*_C-15T	*rpoB_*WT; *katG*_WT; *inhA*_C-15T	ND	19
	*rpoB_*WT; *katG*_S315T1; *inhA*_C-15T	*rpoB_*WT; *katG*_S315T1; *inhA*_C-15T	ND	1
	*rpoB_*WT; *katG*_WT absent [inferred]; *inhA*_WT	*rpoB_*WT; *katG*_S315N; *inhA*_WT	*katG*_S315N, 944 agc>aac	2
		*rpoB_*WT; *katG*_S315R; *inhA*_WT	*katG*_G316del, 946 del ggc	1
	*rpoB_*WT; *katG*_WT; *inhA*_WT2 absent [inferred]	*rpoB_*WT; *katG*_WT; *inhA*_T-8G	*inhA_*T-8G	1
	*rpoB_*WT; *katG*_S315T1+WT [HR]; *inhA*_WT	*rpoB_*WT; ***katG*****_S315T1**; *inhA*_WT	*katG*_S315T1+S315=, 944 agc>acc + 944 agc>= [HR]	1
		*rpoB_*WT; ***katG*****_S315R**; *inhA*_WT	*katG*_S315T1+S315=, 944 agc>acc + 944 agc>= [HR]	1
		*rpoB_*WT; ***katG*****_WT**; *inhA*_C-15T	*katG_*WT	1
	*rpoB_*WT; *katG*_WT; *inhA*_C-15T+WT1 [HR]	*rpoB_*WT; *katG*_WT; ***inhA*****_indeterminate**	*inhA*_C-15T+WT [HR]	1
MDR (*N* = 41)	*rpoB*_D435V; *katG*_S315T1; *inhA*_WT	*rpoB*_Q432L/D435V; *katG*_S315T1; *inhA*_WT	ND	1
	*rpoB_*H445D; *katG*_WT; *inhA*_C-15T	*rpoB_*H445D; *katG*_WT; *inhA*_C-15T	ND	1
	*rpoB_* S450L; *katG*_S315T1; *inhA*_WT	*rpoB_*S450L/W; *katG*_S315T1; *inhA*_WT	ND	15
	*rpoB_* S450L; *katG*_WT; *inhA*_C-15T	*rpoB_*S450L/W; *katG_*WT; *inhA_*C-15T	ND	5
	*rpoB_*S450L+WT8 [HR]/H445Y faint+WT7 [HR]; *katG*_WT; *inhA*_C-15T	***rpoB_* S450L/W**; *katG*_WT; *inhA*_C-15T	*rpoB*_S450L, 1349 tcg>ttg	1
	*rpoB_*S450L; *katG_*WT; *inhA_*C-15T+WT [HR]	*rpoB_* S450L/W; *katG*_WT; ***inhA*_C-15T**	*inhA*_C-15T	1
	*rpoB_*WT3+4 absent [inferred]; *katG_*S315T1; *inhA_*WT	*rpoB*_Q432L/D435V; *katG*_S315T1; *inhA*_WT	*rpoB*_D435V, 1304 gac>gtc	1
	*rpoB_*WT7 absent [inferred]; *katG_*S315T1; *inhA_*WT	*rpoB_*H445C; *katG*_S315T1; *inhA*_WT	*rpoB*_H445C, 1333 cac>tgc	2
		*rpoB_*H445N; *katG*_S315T1; *inhA*_WT	*rpoB*_H445N/D/A/T, 1333 cac>aac/cac>gac/cac>gcc/cac>acc	1
	*rpoB_*WT7 absent [inferred]; *katG_*S315T1; *inhA_*T-8C	*rpoB_*H445L; *katG*_S315T1; *inhA*_T-8C	*rpoB*_H445L, 1334 cac>ctc	1
	*rpoB_*WT8 absent [inferred]; *katG_*S315T1; *inhA_*WT	*rpoB_*L452P; *katG*_S315T1; *inhA*_WT	*rpoB*_L452P, 1355 ctg>ccg	2
	*rpoB_* S450L; *katG_*WT absent [inferred]; *inhA*_WT	*rpoB_*S450L/W; *katG*_S315N; *inhA*_WT	*katG*_S315N, 944 agc>aac	2
	*rpoB_* S450L; *katG_*WT absent [inferred]; *inhA*_C-15T	*rpoB_*S450L/W; *katG*_S315N; *inhA*_C-15T	*katG*_S315N+A379T, 944 agc>aac+1135 gcc>acc	1
	*rpoB_* S450L+WT8 [HR]; *katG*_S315T1+WT [HR]; *inhA*_WT	***rpoB*_WT**; ***katG_*S315R**; inhA_WT	*rpoB_*S450L (1349 tcg>ttg + 1349 tcg>=) [HR]; *katG*_S315T1+S315= (944 agc>aCc + 944 agc>=) [HR]	1
	*rpoB_* S450L+WT8 [HR]; *katG_*WT absent [inferred]; *inhA*_WT	***rpoB_*indeterminate**; *katG*_S315N; *inhA*_WT	*rpoB_*S450L (1349 tcg>ttg + 1349 tcg>=) [HR]; *katG*_S315N, 944 agc>aac	1
	*rpoB_*WT1 absent [inferred]; *katG*_S315T1; *inhA*_WT	***rpoB_*WT**; *katG*_S315T1; *inhA*_WT	*rpoB*_T427A, 1279 acc>gcc; *katG_*S315T1+S315= (944 agc> acc + 944 agc>=) [HR]	1
	*rpoB_*WT3+4+7 absent [inferred]; *katG_*S315T1; *inhA_*C-15T	***rpoB_*indeterminate**; *katG*_S315T1; *inhA*_C-15T	*rpoB*_M434I+H445N, 1302 atg>ata+1333 cac>aac	1
	*rpoB_*WT7 absent [inferred]; *katG_*WT; *inhA_*C-15T+WT [HR]	*rpoB_*H445N; *katG*_WT; ***inhA*_WT**	*rpoB*_H445N, 1333 cac>aac; *inhA*_C-15T+WT [HR]	2
	*rpoB_*WT7 absent [inferred]; *katG_*S315T1; *inhA_*WT1 absent [inferred]	*rpoB_*H445L; *katG*_S315T1; ***inhA*_indeterminate**	*rpoB*_H445L, 1334 cac>ctc; inhA_G-17T	1
MTBC negative (*N* = 34)	TUB band negative	No MTBC DNA detected	ND	30
		***rpoB*_WT**; ***katG*_WT**; ***inhA*_WT**	*M. fortuitum^b^* ^*,* *d*^	1
		***rpoB+katG+inhA_*indeterminate**	*M. doricum^b^*	1
			*M. abscessus* subsp*. abscessus^b^*^,*d*^	1
			*M. gordonae^b^* ^*,* *d*^	1

^*a*^ FluoroType results discordant with the LPA MTBDR*plus* are bolded; ND, not done; HR, heteroresistant.

^*b*^ For non-tuberculous mycobacteria identification, LPAs GenoType Mycobacteria CM and NTM-DR, and, when necessary, PRA-*hsp65 *were used.

^*c*^ Isolate identified as a mixed culture of mycobacteria (MTBC, *M. fortuitum, *and* M. intracellulare*), patient has an Xpert MTB/RIF Ultra susceptible result.

^*d*^ Non-tuberculous mycobacteria isolates from patients diagnosed with and treated for TB.

Among the 44 isolates with *rpoB* mutations detected by LPA, only seven (15.9%) harbored mutations that could be specifically identified by the assay, while the remaining 37 (84.1%) presented inferred resistance patterns based on missing wild-type probes: one WT1-negative, four WT2-negative, five WT3-negative, one WT3+4-negative, one WT5-negative, and 25 WT7-negative (Tables 1 and 3).

FluoroType detected *rpoB* mutations in 42 of the 44 isolates identified as mutated by LPA, yielding a sensitivity of 95.5% (Table 2). Five of these 42 isolates carried the silent *rpoB* F433= mutation and were correctly classified as RIF-susceptible by FluoroType (Tables 1 and 3). Of the two discordant isolates, one showed a WT1-negative pattern by LPA but was classified as *rpoB* indeterminate by FluoroType (silent T427= by sequencing), while the other showed heteroresistance, defined by the simultaneous hybridization of the WT8 wild-type probe (covering the *rpoB* region encompassing codon S450) and the corresponding MUT3 (S450L) probe, but was classified as wild type by both FluoroType and sequencing (Tables 1 and 3).

Additionally, FluoroType identified the H445C mutation in an isolate with an inferred WT5-negative pattern by LPA, a region not specifically targeted by the WT5 probe. Similarly, in another isolate lacking the WT7 band on LPA, FluoroType detected the S441L mutation, which is also not covered by this probe (Table 3). Despite these differences in mutation characterization, these cases were considered concordant, as both methods correctly classified the isolates as RIF-resistant.

Among the 1,762 isolates classified as RIF-susceptible by LPA, 1,758 were concordant by FluoroType, corresponding to a specificity of 99.8%. Overall, the accuracy of FluoroType for RIF resistance detection was 99.7% (Table 2).

One isolate showed wild-type results for both *katG* and *inhA* by LPA, but absence of *rpoB* WT6+7+8 bands—indicative of low DNA load; it was classified as wild type for all genes by FluoroType, in agreement with sequencing (Tables 1 and 3). Interestingly, this isolate was identified as a mixed culture of mycobacteria (MTBC, *M. fortuitum*, and *M. intracellulare*), and the patient had an Xpert MTB/RIF Ultra susceptible result.

Regarding INH resistance, 34 of the 36 INH-resistant isolates identified by LPA were concordant by FluoroType, corresponding to a sensitivity of 94.4% (Table 2). Three isolates exhibited *katG* heteroresistance by LPA (S315T1+WT present). Of these, two were considered concordant by FluoroType: in one isolate, FluoroType detected only the S315T1 mutation and sequencing confirmed heteroresistance; in the other, sequencing also confirmed *katG* heteroresistance, while FluoroType identified the S315R mutation, which was considered concordant with LPA as it is also associated with INH resistance.

For the third heteroresistant isolate, both FluoroType and sequencing classified *katG* as wild type. In addition, one inhA heteroresistant isolate (C-15T+WT1 present) identified by LPA was classified as *inhA* indeterminate by FluoroType but confirmed as heteroresistant by sequencing (Tables 1 and 3).

FluoroType specificity for INH resistance detection was 100%, and overall accuracy was 99.9% (Table 2).

Unlike RIF-R isolates, which showed a high frequency of inferred *rpoB* mutations by LPA, only 12 (29.3%) of the 41 MDR isolates exhibited inferred mutations. Sensitivity of FluoroType for MDR detection in comparison to LPA was 85.4% (35/41). Discordances among MDR isolates were associated with heteroresistance (*n* = 3) and inferred mutations (*n* = 3) on LPA. Among the heteroresistant discordant isolates, one was *rpoB* S450L+WT8 and *katG* S315T1+WT by LPA, but was identified as *rpoB* wild type and *katG* S315R by FluoroType. Sequencing confirmed heteroresistance, and the discordance was considered only for RIF-R detection, as the S315R mutation detected by FluoroType is associated with INH-R. A second isolate, also *rpoB* S450L+WT8 by LPA, tested *rpoB* indeterminate on FluoroType, with sequencing again confirming heteroresistance. The third heteroresistant discordant isolate was *inhA* C-15T+WT1 by LPA, but tested *inhA* wild type on FluoroType, which was confirmed by sequencing (Tables 1 and 3).

Among the three discordant isolates with inferred mutations by LPA, one showed absence of *rpoB* WT1 on LPA, was classified as wild type by FluoroType, and harbored the T427A mutation by sequencing. The remaining two isolates yielded indeterminate results by FluoroType: one with *rpoB* WT3/4/7 absence on LPA and double *rpoB* mutations (M434I+H445N) detected by sequencing, and another with absence of *inhA* WT1 on LPA, confirmed by sequencing to carry the G-17T substitution (Tables 1 and 3). An additional finding was an isolate lacking the WT7 probe on LPA and classified as H445N by FluoroType, in which sequencing revealed a mixed H445N/D/A/T profile, consistent with the presence of multiple mutant subpopulations at codon 445. FluoroType showed a specificity of 100% and an overall accuracy of 99.7% for MDR detection (Table 2).

## DISCUSSION

The FluoroType MTBDR assay represents a new generation of automated, high-throughput molecular platforms for the rapid detection of MTBC and resistance to RIF and INH. In this study, conducted under routine conditions in a high TB burden setting, FluoroType demonstrated excellent performance for MTBC identification, with 100% sensitivity and near-perfect agreement (accuracy of 99.8%) with the LPA. In addition, the assay showed high sensitivity (85.4%–95.5%), specificity (99.8%–100%), and overall accuracy (99.7%–99.9%) for the detection of drug resistance, further supporting its reliability for routine diagnostic use. These findings are consistent with previous evaluations reporting high sensitivity for RIF and INH resistance detection and specificity approaching 100% when compared with LPA or composite reference standards (21, 22).

As a reference laboratory processing mycobacterial cultures, we evaluated FluoroType using a large and diverse collection of clinical isolates, including samples grown in liquid (*n* = 1,608; 87.3%) and solid (*n* = 233; 12.7%) media, with varying bacterial loads and mutation profiles. Despite the overall high performance, four NTM isolates were misclassified as MTBC by FluoroType, and three corresponding patients were diagnosed with and treated for TB. This discordance may reflect the presence of low levels of MTBC DNA in mixed cultures that were not detected by the LPA in these three cases.

Low DNA input may have contributed to the discordant isolate classified as wild type by LPA (and confirmed by sequencing) but yielding an indeterminate *katG* result by FluoroType. Since DNA extraction for LPA was performed first and the remaining material was subsequently used for FluoroType, differences in template availability or uneven distribution of bacilli may have influenced target detection between assays. However, the DNA input was not quantified, and this hypothesis cannot be confirmed. Importantly, repeat FluoroType testing was performed after a new DNA extraction, but the indeterminate *katG* result persisted, suggesting that factors beyond DNA availability may have contributed to the discordance. These findings highlight the possibility of occasional assay-specific limitations, and other factors, including variability in target detection when template abundance is limited, differences in analytical sensitivity, or sequence variations affecting probe hybridization, may also contribute to indeterminate results and warrant further investigation.

In the other discordant isolate identified as wild type by LPA but detected as RIF-R (*rpoB* R448K) by FluoroType, sequencing revealed a P454S mutation located outside the RRDR. The *rpoB* gene’s RRDR, spanning codons 426 to 452, represents the primary hotspot for mutations associated with RIF resistance, and most molecular assays, including LPA and FluoroType, are designed to target this region (6). This finding suggests that variants outside the targeted region may lead to misinterpretation by molecular assays, highlighting limitations in mutation resolution and supporting the use of sequencing to resolve discrepant or ambiguous results, as previously reported (21, 23). Unfortunately, pDST was not available for this isolate because the LPA is the routine method used in our laboratory for detecting RIF and INH resistance.

A high proportion of RIF-resistant isolates showed inferred *rpoB* mutations by LPA (84.1%), predominantly involving the WT7 probe and corresponding to the borderline H445N mutation. The high frequency of this mutation is consistent with previous findings from our setting, which have demonstrated the sustained circulation of clustered MTBC isolates harboring H445N in SP (24, 25). In contrast, only 29.3% of MDR isolates exhibited inferred *rpoB* mutations, suggesting that the mutation profiles of RIF-resistant and MDR isolates may differ, as previously reported, and likely reflect distinct epidemiological and evolutionary pathways (26).

In addition, there were isolates with inferred *rpoB* mutations by LPA that were subsequently identified as synonymous substitutions by sequencing, including T427= (WT1 negative, *n* = 1), F433= (WT3 negative, *n* = 5), and T444= (WT7 negative, *n* = 1). As LPA detects the absence of wild-type probes without distinguishing the underlying nucleotide change, these mutations are interpreted as resistance-associated and reported as RIF-R, representing a known limitation of LPAs. FluoroType correctly identified and classified all F433= isolates as susceptible, unlike those with T444= and T427=, which were misclassified or indeterminate.

The F433= mutation is typically detected as susceptible by the Xpert MTB/RIF Ultra (27), and in our routine practice, the combination of a susceptible Xpert result with WT3 absence in LPA supports its inference and is reported with an interpretative comment. Among five such cases, four were susceptible and one indeterminate by Xpert. In contrast, T444= was classified as RIF-resistant by Xpert, and no Xpert result was available for T427=, highlighting inconsistencies across molecular assays in the presence of synonymous variants.

The discordant results observed in this study are consistent with those reported by Hillemann et al. (23), highlighting the limitations of FluoroType in accurately resolving specific resistance-associated mutations. Among RIF-R/MDR isolates with inferred *rpoB* mutations by LPA (*n* = 49), FluoroType correctly identified most cases (42/49, 85.7%); however, misidentifications were observed, including incorrect mutation assignment (e.g., L430P instead of L430R and H445C instead of P439L), misclassification of a synonymous mutation (T444=) as a resistance-associated variant (S441L), and indeterminate results in isolates with a synonymous mutation (T427=) or double mutations (M434I+H445N). An additional *rpoB* indeterminate result by FluoroType was observed in an isolate with heteroresistance (S450L+WT) by both LPA and sequencing. These findings support previous evidence that melting curve-based approaches may have limited resolution for rare, closely spaced, heteroresistant, or synonymous variants, resulting in false resistance calls or indeterminate outputs.

For INH resistance, most results were concordant, with discrepancies primarily involving heteroresistant isolates and uncommon variants. FluoroType incorrectly identified a *katG* codon 316 deletion as S315R and interpreted heteroresistant patterns (S315T+WT) as S315R in two isolates, as well as yielding indeterminate results for *inhA* mutations, including both heteroresistance (C-15T+WT) and the G-17T substitution. Together, these results indicate that, while FluoroType performs well for resistance detection overall, its ability to accurately identify specific mutations is limited, particularly in the context of detecting minority resistant subpopulations.

LPA, routinely performed in our laboratory, is more labor-intensive than FluoroType and requires multiple rooms for different steps of the procedure. DNA extraction and PCR are conducted manually in batches of 47 isolates plus one negative control. Hybridization of the 48 tests is carried out using the GT-Blot 48 system, with results analyzed using GenoScan. In our laboratory, staff performing the assay work 4 h daily shifts, resulting in a total turnaround time of approximately 3 days for the release of 47 isolates: 1 day for DNA extraction and PCR, 1 day for hybridization, and 1 day for strip drying, reading, and result interpretation (28).

In contrast, FluoroType simplifies the workflow by eliminating the hybridization step and enabling automated amplification and detection. DNA extraction and PCR setup are performed manually, after which amplification and analysis are completed in the FluoroCycler XT instrument with automated result interpretation. Under our routine conditions, the workflow is completed over 2 days: 2 days for DNA extraction of 94 samples and a second day for PCR, analysis, and result release. This allows processing and release of up to 94 isolates within 2 days, effectively doubling throughput while reducing turnaround time compared to LPA.

The prospective evaluation of a large number of clinical isolates under real-world conditions represents a major strength of this study. The inclusion of isolates with diverse mutation patterns, low bacterial loads, and mixed cultures provides a realistic assessment of assay performance.

This study has limitations. First, sequencing was performed only for discrepant or indeterminate results, which may have limited the detection of additional mutations or minority variants. Second, DNA extraction for LPA was performed prior to FluoroType, potentially introducing bias due to unequal bacillary distribution and affecting comparative results.

In conclusion, FluoroType is a rapid, accurate, and high-throughput tool for MTBC detection and first-line drug resistance profiling in cultured isolates. Its implementation in reference laboratories may improve diagnostic efficiency and reduce turnaround time. However, caution is warranted in the interpretation of specific mutations, particularly in cases of heteroresistance, synonymous, or uncommon variants, for which confirmatory testing by sequencing may be required.

## Supplementary Material

Reviewer comments

## Data Availability

The DNA sequences of all the sequenced isolates were deposited in the NCBI Sequence Read Archive (http://www.ncbi.nlm.nih.gov/sra) under accession numbers SRR35854613–SRR35854689, as part of/within Bioproject PRJNA888434.

## SUPPLEMENTAL MATERIAL

OPEN PEER REVIEW reviewer-comments.pdf An accounting of the reviewer comments and feedback.

ASM does not own the copyrights to Supplemental Material that may be linked to, or accessed through, an article. The authors have granted ASM a non-exclusive, world-wide license to publish the Supplemental Material files. Please contact the corresponding author directly for reuse.
