# Supplementary material for: Real-world evaluation of the FluoroType MTBDR 2.0 assay for detection of rifampicin and isoniazid resistance-conferring mutations in Mycobacterium tuberculosis complex clinical isolates
Source: Microbiol Spectr. 2026 Sep 18;14(11):e01880-26. doi: 10.1128/spectrum.01880-26 (PMC13729130; doi:10.1128/spectrum.01880-26)
Supplement: Supplementary file 1 [file reviewer-comments.pdf]

ASM | Microbiology Spectrum

Real-world evaluation of the FluoroType MTBDR 2.0 assay for detection of rifampicin and isoniazid resistance-conferring mutations in *Mycobacterium tuberculosis* complex clinical isolates

Fabiane Ferreira, Angela Brandao, Thiago Massanti, Antonio Rego, Lucilaine Ferrazoli, Erica Chimara, Rosangela de Oliveira, and Juliana Pinhata

Corresponding Author(s): Juliana Pinhata, Instituto Adolfo Lutz

Review Timeline:

Submission Date:	June 5, 2026
Editorial Decision:	July 20, 2026
Revision Received:	August 3, 2026
Accepted:	August 14, 2026

Editor: Kenneth Gavina

Reviewer(s): Disclosure of reviewer identity is with reference to reviewer comments included in decision letter(s). The following individuals involved in review of your submission have agreed to reveal their identity: Sydney Stanley (Reviewer #2)

Transaction Report:

(Note: With the exception of the correction of typographical or spelling errors that could be a source of ambiguity, letters and reports are not edited. The original formatting of letters and referee reports may not be reflected in this compilation.)

DOI: https://doi.org/10.1128/spectrum.01880-26

Re: Spectrum01880-26 (Real-world evaluation of the FluoroType MTBDR 2.0 assay for detection of rifampicin and isoniazid resistance-conferring mutations in *Mycobacterium tuberculosis* complex clinical isolates)

Dear Dr. Juliana Maira Watanabe Pinhata:

Thank you for submitting your manuscript to ASM Microbiology Spectrum. Your manuscript has been reviewed by two experts in the field and the consensus decision is for resubmission with modifications. Below you will find the instructions from the Spectrum editorial office and the reviewer comments. Please ensure to address the reviewer comments completely and comprehensively.

Please return the manuscript within 60 days; if you cannot complete the modification within this time period, please contact me. If you do not wish to modify the manuscript and prefer to submit it to another journal, notify me immediately so that the manuscript may be formally withdrawn from consideration by Spectrum.

Revision Guidelines
To submit your modified manuscript, log into the submission site at https://spectrum.msubmit.net/cgi-bin/main.plex. Go to Author Tasks and click the appropriate manuscript title to begin. The information you entered when you first submitted the paper will be displayed; update this as necessary. Note the following requirements:

• Upload point-by-point responses to the issues raised by the reviewers in a file named "Response to Reviewers," NOT in your cover letter.
• Upload a compare copy of the manuscript (without figures) as a "Marked-Up Manuscript" file.
• Upload a clean .DOC/.DOCX version of the revised manuscript and remove the previous version.
• Each figure must be uploaded as a separate, editable, high-resolution file (TIFF or EPS preferred), and any multipanel figures must be assembled into one file.
• Any supplemental material intended for posting by ASM should be uploaded with their legends separate from the main manuscript. You can combine all supplemental material into one file (preferred) or split it into a maximum of 10 files with all associated legends included.

For complete guidelines on revision requirements, see our Submission and Review Process webpage. Submission of a paper that does not conform to guidelines may delay acceptance of your manuscript.

Data availability: ASM policy requires that data be available to the public upon online posting of the article, so please verify all links to sequence records, if present, and make sure that each number retrieves the full record of the data. If a new accession number is not linked or a link is broken, provide Spectrum production staff with the correct URL for the record. If the accession numbers for new data are not publicly accessible before the expected online posting of the article, publication may be delayed; please contact production staff (Spectrum@asmusa.org) immediately with the expected release date.

Publication Fees: For information on publication fees and which article types are subject to charges, visit our website. If your manuscript is accepted for publication and any fees apply, you will be contacted separately about payment during the production process; please follow the instructions in that e-mail. Arrangements for payment must be made before your article is published.

ASM Membership: Corresponding authors may join or renew ASM membership to obtain discounts on publication fees. Need to upgrade your membership level? Please contact Customer Service at Service@asmusa.org.

The ASM Journals program strives for constant improvement in our submission and publication process. Please tell us how we can improve your experience by taking this quick Author Survey.

Sincerely,
Kenneth Gavina
Editor
Microbiology Spectrum

Reviewer #1 (Comments for the Author):

The authors present a well-rounded study of the FluoroType MTBDR 2.0 assay compared to their current standard of care LPA GenoType MTBDRplus assay. Minor comments and suggestions are below:

Line 55- Add "many" or another word between "shared by" and "moleclar assays", as sequencing is still considered a molecular test

Line 130- is a year missing after January? Since the rate of MDR isolates is low, seems likely isolates were collected over multiple years.

Line 288- add percentage after "near-perfect agreement"

Line 288- please define "high sensitivity" either by giving a range or stating the technical performance

Table 2- move this data to table 3 or table S1 as discrepant analysis and provide a summary table as the new table 2 to include the positive/negative agreement and other test characteristics of the LPA vs the FluoroType assays

Reviewer #2 (Comments for the Author):

This study effectively evaluates the performance of the FluoroType assay in comparison to a LPA. The manuscript is clearly written and does a nice job highlighting the pitfalls of molecular assays and noting limitations to genetic susceptibility calling that should be considered. Table 2 is a strength of the manuscript because this list of all the mutations and discrepancies could be a useful resource to address the gaps in these assays. I have listed minor suggestions to improve the clarity of the communication and to detail some of the mechanisms of the discrepancies, which may be helpful as we look to circumvent the issues.

Lines 36-37: "Among isolates classified as wild-type by LPA, sensitivity was 99.9% (1,683/1,685)"

The wording is confusing, it isn't clear what the sensitivity is evaluating. I would rephrase along the lines of "FluoroType demonstrated 99.9% (1,683/1,685) sensitivity in detecting wild-type isolates"

Line 38: MDR abbreviation should be spelled out

Lines 48-50: "This study evaluates the performance of the FluoroType MTBDR assay for detecting Mycobacterium tuberculosis complex (MTBC) and resistance to rifampicin and isoniazid under routine conditions in a high-burden tuberculosis (TB) reference laboratory."

I would emphasize the novelty of this study in the "Importance" section. Is this study the first to evaluate the assay under these conditions? How does it differ from others?

Lines 90-94: "Several of these technologies are currently recommended by the World Health Organization (WHO) for the initial diagnosis of TB and rapid detection of resistance to RIF and INH directly from pulmonary specimens(6). The FluoroType MTBDR assay (FluoroType) (Bruker-Hain Lifescience, Germany) is one such WHO-endorsed technology."

Similar to above, I would highlight how the FluoroType assay in particular compares to other PCR assays

Line 153: TUB abbreviation should be spelled out

Lines 181-183: "Following the methodology described by Pinhata et al.(16), isolates showing discordant results between FluoroType and the LPA, as well as those with inferred resistance-associated mutations identified by the LPA, were subjected to Sanger sequencing."

"inferred resistance-associated mutations" should be defined

Lines 229-233: "Of the two discordant isolates, one showed a WT1-negative pattern by LPA but was classified as rpoB indeterminate by FluoroType (silent T427= by sequencing), while the other was heteroresistant by LPA (S450L + WT8 present) but classified as wild-type by both FluoroType and sequencing (Tables 1 and 2)."

Does WT8 refer to the WT allele at the 450 codon? This should be clarified somewhere in the text. The term "heteroresistance" should be defined by the study, I think it is commonly defined a mix of resistant and susceptible populations within a sample, here it seems like it is defined by a sample having two different alleles which are not necessarily the same thing if WT8 does not refer to codon 450 WT allele.

Lines 219-221: "and another in which FluoroType detected an rpoB mutation (R448K), whereas sequencing indicated a wild-type RRDR, although a P454S mutation located outside the RRDR was identified (Tables 1 and 2).'

Was this strain phenotypically susceptible or resistant? maybe that should be the tiebreaker for the funcationality of mutations outside the RRDR

Line 222: "Among the 44 isolates with rpoB mutations detected by LPA alone"

Shouldn't this just read as "Among the 44 isolates with rpoB mutations detected by LPA"?

Lines 260-261: "Unlike RIF-R isolates, which showed a high frequency of inferred rpoB mutations by LPA, only 12 (29.3%) of the 41 MDR isolates exhibited inferred mutations."

Anythought as to whyvthis is the case?

Lines 300-303: "Low DNA input may explain a discordant isolate classified as wild-type by LPA (and confirmed by sequencing), but as katG indeterminate by FluoroType. DNA extraction for LPA was performed first, and the remaining material was subsequently used for FluoroType, which may have contributed to uneven distribution of bacilli and differential detection between assays."

This is not a very convincing explanation, what happens when you repeat with higher DNA input? Was the input actually lower? Are there any other hypotheses explaining the difference?

Table 2: It may be difficult to incorporate in the table but it would be nice to have the FluoroType results included somewhere to focus muations for those where susceptibility calls were different. Could be highlighted in Table 2 or made into another table.

<u>**Response to reviewers**</u>

Manuscript ID: Spectrum01880-26 (Real-world evaluation of the FluoroType MTBDR 2.0 assay for detection of rifampicin and isoniazid resistance-conferring mutations in *Mycobacterium tuberculosis* complex clinical isolates)

We are grateful to the reviewers for their thorough assessment of our manuscript and for their valuable comments and suggestions. Their feedback has contributed significantly to improving the presentation and interpretation of our findings. We have carefully addressed each point raised and have revised the manuscript where appropriate. Detailed point-by-point responses are provided below, with corresponding changes highlighted in the revised version of the manuscript.

Reviewer #1 (Comments for the Author):

The authors present a well-rounded study of the FluoroType MTBDR 2.0 assay compared to their current standard of care LPA GenoType MTBDR*plus* assay. Minor comments and suggestions are below:

Line 55- Add "many" or another word between "shared by" and "moleclar assays", as sequencing is still considered a molecular test

Response: The Importance section was rewritten after the reviewers' suggestions (lines 51-64).

Line 130- is a year missing after January? Since the rate of MDR isolates is low, seems likely isolates were collected over multiple years.

Response: We thank the reviewer for this comment. No year was missing in this sentence. The MDR isolates included in this analysis were collected between January and December 2024, corresponding to 41 isolates (line 144).

Line 288- add percentage after "near-perfect agreement"

Response: We added '(accuracy of 99.8%) after "near-perfect agreement" (line 314).

Line 288- please define "high sensitivity" either by giving a range or stating the technical performance

Response: We included the ranges for sensitivity, specificity and accuracy (lines 315-316).

Table 2- move this data to table 3 or table S1 as discrepant analysis and provide a summary table as the new table 2 to include the positive/negative agreement and other test characteristics of the LPA vs the FluoroType assays

Response: We have included a new Table 2 summarizing the performance characteristics of FluoroType in comparison with LPA. The previous Table 2, which presents the detailed discrepant analysis and mutation profiles, has been moved and is now presented as Table 3.

Reviewer #2 (Comments for the Author):

This study effectively evaluates the performance of the FluoroType assay in comparison to a LPA. The manuscript is clearly written and does a nice job highlighting the pitfalls of molecular assays and noting limitations to genetic susceptibility calling that should be considered. Table 2 is a strength of the manuscript because this list of all the mutations and discrepancies could be a useful resource to address the gaps in these assays. I have listed minor suggestions to improve the clarity of the communication and to detail some of the mechanisms of the discrepancies, which may be helpful as we look to circumvent the issues.

Lines 36-37: "Among isolates classified as wild-type by LPA, sensitivity was 99.9% (1,683/1,685)" The wording is confusing, it isn't clear what the sensitivity is evaluating. I would rephrase along the lines of "FluoroType demonstrated 99.9% (1,683/1,685) sensitivity in detecting wild-type isolates"

Response: We have modified the text according to the suggestion (lines 38-39).

Line 38: MDR abbreviation should be spelled out

Response: MDR was spelled out (line 40).

Lines 48-50: "This study evaluates the performance of the FluoroType MTBDR assay for detecting Mycobacterium tuberculosis complex (MTBC) and resistance to rifampicin and isoniazid under routine conditions in a high-burden tuberculosis (TB) reference laboratory."

I would emphasize the novelty of this study in the "Importance" section. Is this study the first to evaluate the assay under these conditions? How does it differ from others?

Response: We have changed the Importance section accordingly (lines 51-64).

Lines 90-94: "Several of these technologies are currently recommended by the World Health Organization (WHO) for the initial diagnosis of TB and rapid detection of resistance to RIF and INH directly from pulmonary specimens(6). The FluoroType MTBDR assay (FluoroType) (Bruker-Hain Lifescience, Germany) is one such WHO-endorsed technology."
Similar to above, I would highlight how the FluoroType assay in particular compares to other PCR assays
Response: FluoroType features were highlighted (lines 102-117).

Line 153: TUB abbreviation should be spelled out
Response: TUB was spelled out (line 167).

Lines 181-183: "Following the methodology described by Pinhata et al.(16), isolates showing discordant results between FluoroType and the LPA, as well as those with inferred resistance-associated mutations identified by the LPA, were subjected to Sanger sequencing."
"inferred resistance-associated mutations" should be defined
Response: Inferred resistance-associated mutations were defined (lines 202-203).

Lines 229-233: "Of the two discordant isolates, one showed a WT1-negative pattern by LPA but was classified as rpoB indeterminate by FluoroType (silent T427= by sequencing), while the other was heteroresistant by LPA (S450L + WT8 present) but classified as wild-type by both FluoroType and sequencing (Tables 1 and 2)."
Does WT8 refer to the WT allele at the 450 codon? This should be clarified somewhere in the text. The term "heteroresistance" should be defined by the study, I think it is commonly defined a mix of resistant and susceptible populations within a sample, here it seems like it is defined by a sample having two different alleles which are not necessarily the same thing if WT8 does not refer to codon 450 WT allele.
Response: Yes, WT8 refers to the WT allele at the 450 codon and it was clarified in the text (lines 254-257). Heteroresistance was defined in lines 172-174, under 'GenoType MTBDR*plus* LPA' section.

Lines 219-221: "and another in which FluoroType detected an rpoB mutation (R448K), whereas sequencing indicated a wild-type RRDR, although a P454S mutation located outside the RRDR was

identified (Tables 1 and 2).'
Was this strain phenotypically susceptible or resistant? maybe that should be the tiebreaker for the functionality of mutations outside the RRDR
Response: Unfortunately, phenotypic drug susceptibility testing (pDST) was not available for this isolate, as the LPA is the routine method used in our laboratory for the detection of rifampicin and isoniazid resistance. In our workflow, pDST is performed only for isolates showing discordant results or when further investigation is required. In this case, the isolate was classified as rifampicin-susceptible by LPA, and the mutation identified outside the rifampicin resistance-determining region (RRDR) was further investigated only in the context of this study; therefore, pDST was not performed. This information has been added to lines 347–349.

Line 222: "Among the 44 isolates with rpoB mutations detected by LPA alone"
Shouldn't this just read as "Among the 44 isolates with rpoB mutations detected by LPA"?
Response: The proposed change was made (line 244).

Lines 260-261: "Unlike RIF-R isolates, which showed a high frequency of inferred rpoB mutations by LPA, only 12 (29.3%) of the 41 MDR isolates exhibited inferred mutations."
Anythought as to why this is the case?
Response: Thank you for this insightful comment. We agree that this is an interesting finding. Studies have shown that rifampicin-monoresistant (RR) and multidrug-resistant (MDR) *Mycobacterium tuberculosis* isolates differ significantly in their *rpoB* mutation profiles, suggesting distinct evolutionary and epidemiological pathways. Consistent with these observations, previous studies from our group have demonstrated a high frequency of inferred *rpoB* mutations among rifampicin-resistant isolates in São Paulo State (24,25). In contrast, inferred *rpoB* mutations appear to be considerably less common among MDR isolates. Although the underlying reasons remain unclear, this difference may reflect the distinct molecular epidemiology of RR and MDR strains circulating in our setting. We are currently conducting whole-genome sequencing studies to investigate the genetic background, transmission dynamics, and evolutionary mechanisms underlying this pattern. We have added this point to the Discussion (lines 350-357).

Lines 300-303: "Low DNA input may explain a discordant isolate classified as wild-type by LPA (and confirmed by sequencing), but as katG indeterminate by FluoroType. DNA extraction for LPA was

performed first, and the remaining material was subsequently used for FluoroType, which may have contributed to uneven distribution of bacilli and differential detection between assays." This is not a very convincing explanation, what happens when you repeat with higher DNA input? Was the input actually lower? Are there any other hypotheses explaining the difference?
Response: We thank the reviewer for this important comment. We agree that the hypothesis of low DNA input is not fully supported by the available data, particularly because DNA concentration was not measured. Moreover, repeat FluoroType testing following a fresh DNA extraction yielded the same indeterminate *katG* result, suggesting that factors other than DNA quantity contributed to the discrepancy. Therefore, we have revised the Discussion to present low DNA input only as a possible, unconfirmed explanation and to emphasize alternative hypotheses, including assay-specific limitations, differences in analytical sensitivity, and sequence variations that may affect probe hybridization (lines 327-339).

Table 2: It may be difficult to incorporate in the table but it would be nice to have the FluoroType results included somewhere to focus muations for those where susceptibility calls were different. Could be highlighted in Table 2 or made into another table.
Response: Thank you for this suggestion. The FluoroType results are already included in Table 3 (former Table 2), in the third column. Therefore, no additional changes were made, as the information requested is already available in the table.

Re: Spectrum01880-26R1 (Real-world evaluation of the FluoroType MTBDR 2.0 assay for detection of rifampicin and isoniazid resistance-conferring mutations in *Mycobacterium tuberculosis* complex clinical isolates)

Dear Dr. Juliana Maira Watanabe Pinhata:

I am delighted to share that your manuscript has been accepted, and I am forwarding it to the ASM production staff for publication. Your paper will first be checked to make sure all elements meet the technical requirements. ASM staff will contact you if anything needs to be revised before copyediting and production can begin. Otherwise, you will be notified when your proofs are ready to be viewed.

Data Availability: ASM policy requires that data be available to the public upon online posting of the article, so please verify all links to sequence records, if present, and make sure that each number retrieves the full record of the data. If a new accession number is not linked or a link is broken, provide production staff with the correct URL for the record. If the accession numbers for new data are not publicly accessible before the expected online posting of the article, publication may be delayed; please contact ASM production staff immediately with the expected release date.

Publication Fees: For information on publication fees and which article types have charges, please visit our website. We have partnered with Copyright Clearance Center (CCC) to collect author charges. If fees apply to your paper, you will receive a message from no-reply@copyright.com with further instructions. For questions related to paying charges through RightsLink, please contact CCC at ASM_Support@copyright.com or toll free at +1-877-622-5543. CCC makes every attempt to respond to all emails within 24 hours.

ASM Membership: Corresponding authors may join or renew ASM membership to obtain discounts on publication fees. Need to upgrade your membership level? Please contact Customer Service at Service@asmusa.org.

Embargo Policy: A press release may be issued as soon as the manuscript is posted on the Spectrum Latest Articles webpage. The corresponding author will receive an email with the subject line "ASM Journals Author Services Notification" when the article is available online.

Cover Image Submissions: If you would like to submit a potential Cover Image, please email a file and a short legend to spectrum@asmusa.org. Please note that we can only consider images that (i) the authors created or own and (ii) have not been previously published. By submitting, you agree that the image can be used under the same terms as the published article. Image File requirements: TIF/EPS, 7.5 inches wide by 8.25 inches tall (at least 2,250 pixels wide by 2,475 pixels tall), minimum 300 dpi resolution (600 dpi preferred), RGB, and no figure elements, e.g., arrows or panel labels. The legend should be a short description of the image, 1-2 sentences recommended. Please use the ASM Journals Cover Image Sizing Tool (https://journals.asm.org/pb-assets/html/asm-cover-sizing-tool.html) to ensure that your proposed cover image meets our size requirements.

The ASM Journals program strives for constant improvement in our submission and publication process. Please tell us how we can improve your experience by taking this quick Author Survey.

Thank you for submitting your paper to Spectrum.

Sincerely,
Kenneth Gavina
Editor
Microbiology Spectrum
